# Prospective phase II trial of [^68^Ga]Ga-NOTA-AE105 uPAR-PET/MRI in patients with primary gliomas: Prognostic value and Implications for uPAR-targeted Radionuclide Therapy

**DOI:** 10.1186/s13550-024-01164-9

**Published:** 2024-10-29

**Authors:** Aleena Azam, Sorel Kurbegovic, Esben Andreas Carlsen, Thomas Lund Andersen, Vibeke André Larsen, Ian Law, Jane Skjøth-Rasmussen, Andreas Kjaer

**Affiliations:** 1 Department of Clinical Physiology and Nuclear Medicine, Copenhagen University Hospital - Rigshospitalet, Blegdamsvej 9, Copenhagen, DK- 2100 Denmark; 2 Cluster for Molecular Imaging, Department of Biomedical Sciences, Copenhagen University Hospital – Rigshospitalet, University of Copenhagen, Copenhagen, Denmark; 3 Department of Neurosurgery, Neuroscience Center, Copenhagen University Hospital – Rigshospitalet, Copenhagen, Denmark; 4 Department of Radiology, Rigshospitalet, Copenhagen University Hospital, Copenhagen, Denmark

**Keywords:** Glioma, Urokinase plasminogen activator receptor (uPAR), Molecular imaging, PET/MRI, Prognosis, Targeted radionuclide therapy

## Abstract

**Background:**

Treatment of patients with low-grade and high-grade gliomas is highly variable due to the large difference in survival expectancy. New non-invasive tools are needed for risk stratification prior to treatment. The urokinase plasminogen activator receptor (uPAR) is expressed in several cancers, associated with poor prognosis and may be non-invasively imaged using uPAR-PET. We aimed to investigate the uptake of the uPAR-PET tracer [^68^Ga]Ga-NOTA-AE105 in primary gliomas and establish its prognostic value regarding overall survival (OS), and progression-free survival (PFS). Additionally, we analyzed the proportion of uPAR-PET positive tumors to estimate the potential number of candidates for future uPAR-PRRT.

**Methods:**

In a prospective phase II clinical trial, 24 patients suspected of primary glioma underwent a dynamic 60-min PET/MRI following the administration of approximately 200 MBq (range: 83–222 MBq) [^68^Ga]Ga-NOTA-AE105. Lesions were considered uPAR positive if the tumor-to-background ratio, calculated as the ratio of TumorSUVmax-to-Normal-BrainSUVmean tumor-SUVmax-to-background-SUVmean, was ≥ 2.0. The patients were followed over time to assess OS and PFS and stratified into high and low uPAR expression groups based on TumorSUVmax.

**Results:**

Of the 24 patients, 16 (67%) were diagnosed with WHO grade 4 gliomas, 6 (25%) with grade 3, and 2 (8%) with grade 2. Two-thirds of all patients (67%) presented with uPAR positive lesions and 94% grade 4 gliomas. At median follow up of 18.8 (2.1–45.6) months, 19 patients had disease progression and 14 had died. uPAR expression dichotomized into high and low, revealed significant worse prognosis for the high uPAR group for OS and PFS with HR of 14.3 (95% CI, 1.8-112.3; *P* = 0.011), and HR of 26.5 (95% CI, 3.3–214.0; *P* = 0.0021), respectively. uPAR expression as a continuous variable was associated with worse prognosis for OS and PFS with HR of 2.7 (95% CI, 1.5–4.8; *P* = 0.0012), and HR of 2.5 (95% CI, 1.5–4.2; *P* = 0.00073), respectively.

**Conclusions:**

The majority of glioma patients and almost all with grade 4 gliomas displayed uPAR positive lesions underlining the feasibility of ^68^Ga-NOTA-AE105 PET/MRI in gliomas. High uPAR expression is significantly correlated with worse survival outcomes for patients. Additionally, the high proportion of uPAR positive gliomas underscores the potential of uPAR-targeted radionuclide therapy in these patients.

**Trail Registration:**

EudraCT No: 2016-002417-21; the Scientific Ethics Committee: H-16,035,303; the Danish Data Protection Agency: 2012-58-0004; clinical trials registry: NCT02945826, 26Oct2016, URL: https://classic.clinicaltrials.gov/ct2/show/NCT02945826.

**Supplementary Information:**

The online version contains supplementary material available at 10.1186/s13550-024-01164-9.

## Introduction

Gliomas are among the most common types of brain cancers, with an annual incidence of 6 cases per 100,000 individuals [1]. These highly heterogenous tumors are graded in a layered approach into 4 distinct WHO grades. Grade 1–2 gliomas are referred to as low-grade (LGG) while grade 3–4 tumors are referred to as high-grade gliomas (HGG). Increasing WHO grade is correlated with increased tumor aggressiveness and poorer survival [2–4]. In the era of many oncological advances, survival among patients with gliomas remains essentially unchanged with a 5-year survival rate of 82% for LGG down to 3% among patients with HGG [4–6]. The treatment of gliomas is highly variable depending on tumor subtype. For LGGs treatment varies from watchful waiting after surgery (biopsy, partial, or gross total resection) to radiotherapy alone or concomitant chemotherapy including a procarbazine, lomustine, vincristine regimen (PCV) or temozolomide (TMZ). For HGG, treatment aims at gross total tumor resection followed by concomitant radiotherapy and chemotherapy with TMZ or PCV [3]. This variability in treatment regimens underlines the need for phenotyping and risk stratification of gliomas before treatment initiation in order to ensure more precise management of these tumors.

Magnetic resonance imaging (MRI) is the standard imaging modality to detect gliomas and can be complemented by positron emission tomography (PET), where particularly the use of amino acid tracers, such as *O*-(2-[^18^F]fluoroethyl)-L-tyrosine (FET), has been recommended [7–9]. FET-PET has multiple applications, including diagnosis, prognostication, target delineation, and determination of tumor recurrence [9]. Additionally, PET imaging with the tracer [^68^Ga]Ga-NOTA-Asp-Cha-Phe-D-Ser-D-Arg-Tyr-Leu-Trp-Ser-OH (^68^Ga-NOTA-AE105) targeting the proteolytic urokinase plasminogen activator (uPA) system is emerging as a promising new imaging biomarker for diagnosis, prognostication, and risk stratification, as well as a therapeutic target for solid cancers [10]. Over the years, several studies have shown the applicability of uPA receptor (uPAR) as a diagnostic biomarker in cancer associated with poor disease prognosis [10]. uPAR is highly upregulated in most solid cancers with limited expression in normal tissue. It is located on the surface of the cell where it binds the serine protease uPA. This facilitates cell proliferation, angiogenesis, proteolysis, and motility resulting in tumor progression and invasion into the surrounding tissue [10–12].

To target uPAR, we developed the PET radiotracer ^68^Ga-NOTA-AE105, where the targeting peptide is a high-affinity antagonist for uPAR [13–15]. We have previously established the safety, biodistribution, and radioligand accumulation of ^68^Ga-NOTA-AE105 in cancer tissue in a Phase 1 trial involving primary tumors and metastases. Tracer accumulation was histopathologically confirmed to correspond with cancer tissue and uPAR expression using immunohistochemistry [13]. Furthermore, we have demonstrated the utility of ^68^Ga-NOTA-AE105 for uPAR-PET as a promising method for noninvasive evaluation of localized prostate cancer with high diagnostic accuracy in differentiating between low-risk and intermediate-risk Gleason score profiles [16]. We have also found uPAR-PET to be highly prognostic in neuroendocrine neoplasms [17], and head-and-neck cancer [18]. In gliomas, we have highlighted uPAR-PET as an effective imaging biomarker for tumor visualization using an orthotopic human xenograft model of glioblastoma [19].

From a therapeutic perspective, we have identified uPAR as a promising target for peptide receptor radionuclide therapy (PRRT) and our team has previously demonstrated the therapeutic efficacy of uPAR-targeted PRRT in preclinical models of prostate and colorectal cancers [20, 21]. Moreover, our recent work has revealed a high correlation between uPAR expression on uPAR-PET and both overall survival (OS) and progression-free survival (PFS) in patients with neuroendocrine neoplasms underscoring uPAR as a promising target for PRRT treatment. In fact, 68% of these patients across tumor grades were uPAR positive [17]. As a result, we hypothesize that uPAR-PET could potentially serve as a prognostic marker of tumor aggressiveness in gliomas and that uPAR-PET positive gliomas may be future candidates for uPAR-targeted PRRT.

Thus, the aim of this prospective phase II clinical trial with ^68^Ga-NOTA-AE105 PET/MRI in patients with primary gliomas was to investigate the association between the uptake of ^68^Ga-NOTA-AE105 on uPAR-PET and both OS and PFS. Furthermore, we aimed to determine the proportion of uPAR-PET positive tumors to assess how many of these patients could potentially be eligible for future uPAR-PRRT.

## Methods

### Study Design

We adhered to the STROBE (Strengthening the Reporting of Observational Studies in Epidemiology) guidelines for reporting. In this prospective clinical trial, eligible patients were enrolled from the Department of Neurosurgery at Copenhagen University Hospital, Rigshospitalet, between March 2017 and June 2022. Patients were eligible if they met the following inclusion criteria: more than 18 years of age, able to read and understand the patient information in Danish and give informed consent, had a newly diagnosed intracranial lesion suspected of primary glioma on brain MRI, and were scheduled for neurosurgery (biopsy or tumor resection).

Patients were excluded if they were pregnant or breastfeeding, had a body weight above 140 kg, had claustrophobia, were above 85 years of age, or suspected of allergy to ^68^Ga-NOTA-AE105.

If the patients were deemed eligible, written informed consent was obtained prior to a preopereative ^68^Ga-NOTA-AE105 PET/MRI brain scan.

### PET/MRI Acquisition

The tracer ^68^Ga-NOTA-AE105 was synthesized as previously described [13]. PET/MRI scan with the radiotracer was performed using an integrated PET/MRI system (Siemens Biograph mMR; Siemens Healthcare). The PET/MRI scan was performed as a dynamic 60-min scan after injection of approximately 200 MBq (median: 202; range: 83–222 MBq) ^68^Ga-NOTA-AE105.

If the patients were not eligible for a PET/MRI scan due to contraindications, a PET/CT scan was performed using a Biograph 128 mCT PET/CT device (Siemens Medical Solutions) with an axial field of view of 21.6 cm. However, of the 24 patients available for final analysis, only 1 patient had undergone PET/CT instead of PET/MRI (see below).

PET images were reconstructed using a Deep Learning-based pseudoCT [22] attenuation map based on a UTE MRI sequence with absolute scatter correction (3-dimensional ordinary Poisson–ordered-subset expectation maximization [3D-OP-OSEM], 4 iterations, 21 subsets, 3.5 mm Gaussian filter). Static images were reconstructed using data acquired from 20 to 40 min, 40–60 min along with a dynamic 0–60 min series following injection of ^68^Ga-NOTA-AE105. The reconstructed PET MRI images 20–40 min following tracer injection were used for further interpretation, quantification, and analysis.

### MRI Protocol

The MRI scan protocol included a UTE AC sequence, a 3D T1-weighted (T1W) MPRAGE both pre- and post-contrast injection with gadolinium, a T2-weighted (T2W) dark-fluid turbo inversion recovery magnitude (TIRM) (FLAIR) in both axial and coronal planes, a diffusion-weighted (DWI) RESOLVE, and a T2W BLADE. Parameters are listed in Table 1.

**Table 1:** MR parameters

MRI sequence	Repetition time (TR) [ms]	Echo time (TE) [ms]	Voxel size
T1W MPRAGE (+/-Gd)	1,900	2.52	1 × 1 × 1mm3
T2W FLAIR	9,000	85	0.69 × 0.69 × 4 mm3
T2W BLADE	5,550	117	0.7 × 0.7 × 5 mm3
DWI	5,600	63	1.2 × 1.2 × 4 mm3
UTE	4.6	0.07/2.46	1.6 × 1.6 × 1.6mm3

### Image Analysis

The analysis of the reconstructed image data was performed independently by a board-certified specialist in nuclear medicine and a board-certified specialist in neuroradiology. Each specialist was blinded to the clinical data. Tumors were delineated by drawing VOIs on the PET images and measured as maximum standardized uptake values (SUVmax). If no uPAR positive lesion was visible on the PET image, the MRI or CT image was used to delineate the tumors for SUVmax measurement. Reference brain VOIs running parallel to the cortex were drawn on the contralateral normal brain hemisphere at a single slice at the level of centrum semiovale. The VOIs were displaced approximately 7 mm from the cortical edge to avoid blood pool activity spill-in and mean standardized uptake value (SUVmean) was measured. A lesion was considered uPAR positive if TumorSUVmax-to-Normal-BrainSUVmean ratio (TBR) was at least 2.0 as used in a previous uPAR-PET study [17]. Tumor size was measured on axial T2W FLAIR MRI images or axial CT images as the product of the maximal perpendicular diameters, according to the Response Assessment in Neuro-Oncology (RANO) criteria [23]. If no lesion was visible on the CT image, the previous MRI scan closest to the PET/CT scan was selected for tumor size measurement.

### Followup

The patients were followed routinely at the Department of Oncology at Copenhagen University Hospital, Rigshospitalet. The follow-up regimen was standardized according to the national Danish glioma guidelines published by the Danish Neuro-Oncological Group (DNOG) [24]. Final follow-up for endpoints was performed October 10, 2023. PFS was evaluated using the RANO criteria and defined as the time from uPAR-PET/MRI scan to progression [23, 25]. OS was defined as the time from uPAR-PET/MRI scan to the time of death. If there was no progression at the time of follow-up, the patient was censored according to the date of the most recent clinical follow-up visit.

### Statistical Analysis

Sample size was calculated based on OS. A total of 29 patients were required in order to detect significant differences (risk of type I error of 0.05 and power of 0.8) in OS at an expected hazard ratio (HR) of 3, a median OS of 14 months, and inclusion period of 12 months and a follow-up time of 36 months. Accounting for potential dropouts 30–35 patients were planned for enrollment in the trial. All continuous variables are reported as mean values with standard deviation (SD) or median with range. In order to compare TumorSUVmax values assessed on the static images 20–40 min and 40–60 min after tracer injection, a spaghetti plot, Bland-Altman analysis with 95% limits of agreement, and percentage agreement, as well as paired t-test analysis were performed. Kaplan-Meier analyses with Cox Proportional-Hazards Regression and log-rank test were performed for PFS and OS estimation and comparison of these between groups, and inverse Kaplan-Meier for median follow-up time. Univariate Cox regression analysis was performed for OS and PFS with uPAR SUVmax as a continuous variable. To establish the optimal cutoff for uPAR SUVmax 20–40 min after tracer injection, we used the Cutoff Finder application [26]. P values less than 0.05 were considered statistically significant. R, version 4.2.2 (R Foundation for Statistical Computing) was used for data analysis.

## Results

### Patients and Image Acquisition

**Fig. 1:**
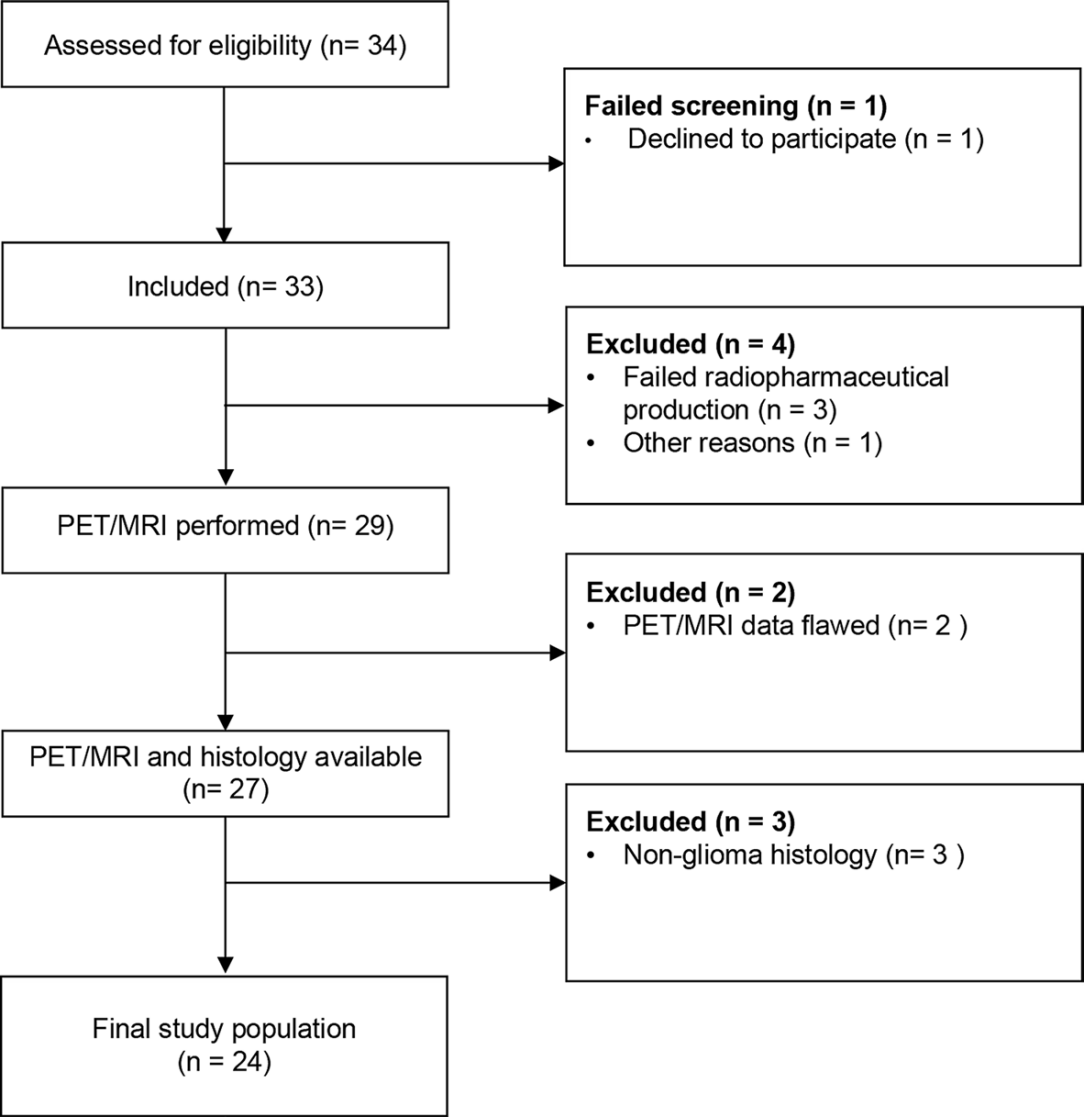
Consolidated standards of reporting trials (CONSORT) flow diagram of inclusion process

A total of 33 patients were enrolled in the trial between March 2017 and June 2022. Out of these, 29 patients underwent imaging with a dynamic PET/MRI (*n* = 26) or PET/CT (*n* = 3) brain scan. Four patients were excluded due to failed radiopharmaceutical production (*n* = 3) and technical issues (*n* = 1). Data was available for reconstruction from 27 of these patients. Histology was available for all 27 patients and was reviewed in accordance with the 2021 WHO classification of central nervous system diseases [2]. Three patients were excluded as they were diagnosed with central nervous system lymphoma. The final trial population thus constituted of 24 patients diagnosed with primary glioma where 23 patients underwent a PET/MRI scan, and 1 patient underwent PET/CT scan, see Fig. 1.

**Fig. 2:**
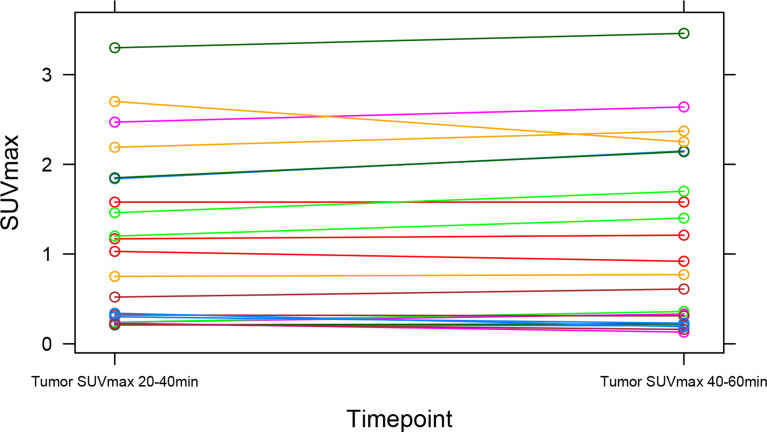
Spaghetti plot illustrating individual trajectories of TumorSUVmax values measured at timepoints 20–40 min and 40–60 min after tracer injection. Each line represents a unique participant (*N* = 22). The plot reveals consistent TumorSUVmax values over time across the study population, emphasizing stability in the measurements over time

**Fig. 3:**
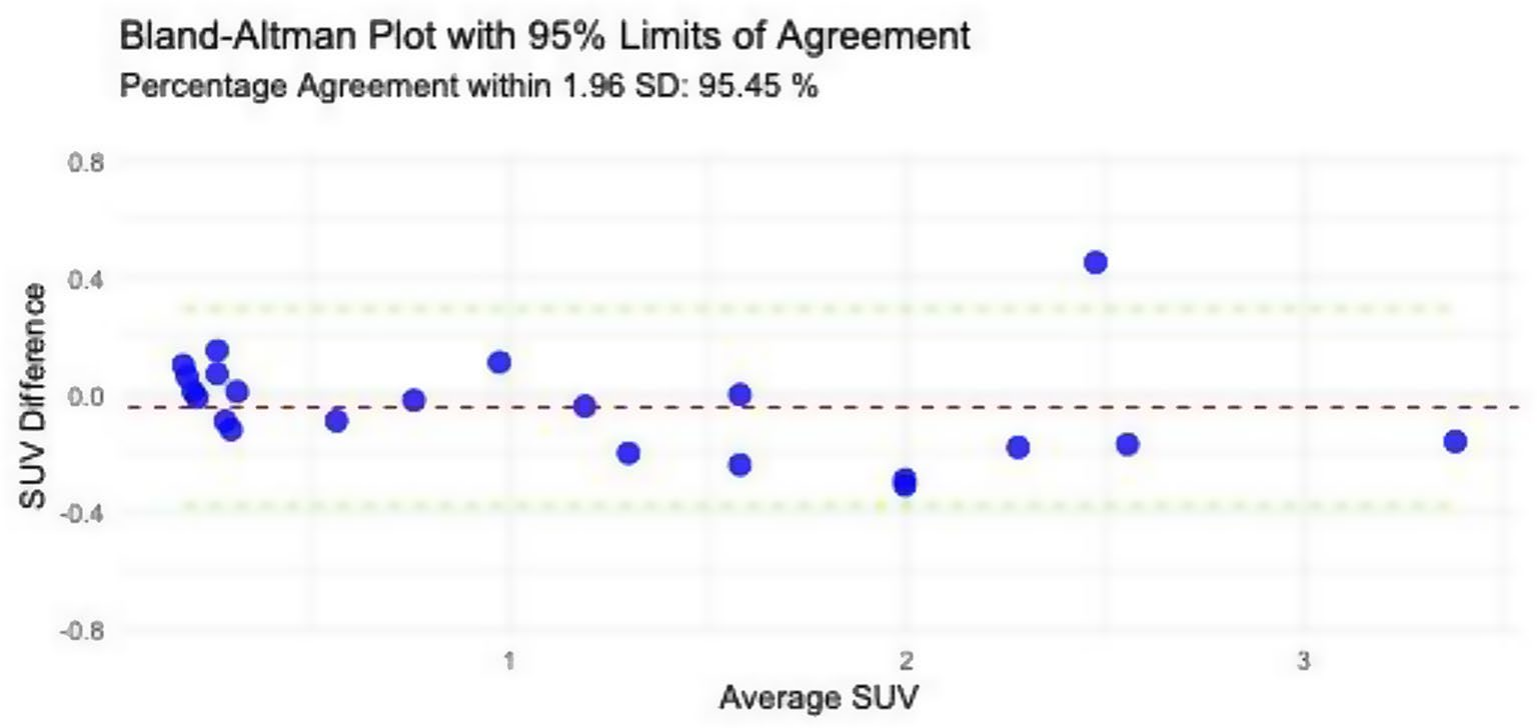
Bland-Altman plot displaying the agreement between TumorSUVmax values measured at timepoints 20–40 min and 40–60 min after tracer injection. The red line represents the mean difference, and the green lines indicate the 95% limits of agreement calculated as mean difference +/- 1.96 SD. Percentage agreement within 1.96 standard deviations is 95.45%, demonstrating high concordance between the two timepoints

The spaghetti plot and Bland-Altman plot of TumorSUVmax values assessed on the static images at time 20–40 and 40–60 min after tracer injection showed good agreement (percentage agreement within 1.96 SD: 95.45%) between the two timepoints, see Figs. 2 and 3. This was verified by the paired t-test, showing no significant difference between the SUVmax values measured at static images from the two different timepoints, mean difference − 0.044 (95% CI, -0.12-0.03; *P* = 0.24). Images at timepoint 40–60 min were missing from 2 patients, as these were not reconstructed due to short image acquisition time. Hence, further interpretation, quantification, and analysis reported is based on the static images at 20–40 min after tracer injection, while the analysis on the static images at 40–60 min after tracer injection is reported in the supplementary material.

Demographic data from the 24 patients is summarized in Table 2. The majority of the patients were diagnosed with WHO grade 4 gliomas (67%, 16/24), followed by grade 3 (25%, 6/24), and grade 2 (8%, 2/24). Most tumors were located in the corpus callosum (21% (5/24), the frontal lobe (25%, 6/24) or the temporal lobe (21%, 5/24). No patients were worse than WHO performance status 1. The median tumor size was 1,700 mm^2^ (range: 320-3,220 mm^2^). The median time from PET/MRI scan to surgery was 1 day (range, 0–21 days). The median injected dose of the tracer ^68^Ga-NOTA-AE105 was 5.0 mL (range, 0.3 mL-7.5 mL), and the median activity was 202 MBq (range, 83–222 MBq). No related adverse events or serious adverse events were recorded during the trial period.

**Table 2:** Baseline Characteristics of Patients with Primary Glioma (*n* = 24)

	**Overall** **(*N*** **= 24)**
**Age (y)**	
Mean (SD)	59.5 (18.1)
Median [Min, Max]	63.5 [22.9, 89.1]
**Sex**	
Female	12 (50.0%)
Male	12 (50.0%)
**Tumor location**	
Corpus callosum	5 (20.8%)
Frontal	6 (25.0%)
Frontoparietal	1 (4.2%)
Insula	1 (4.2%)
Occipital	2 (8.3%)
Parietal	2 (8.3%)
Temporal	5 (20.8%)
Temporoparietal	2 (8.3%)
**Tumor size (mm2)**	
Mean (SD)	1660 (838)
Median [Min, Max]	1700 [320, 3220]
**MRI contrast enhancement**	
No	8 (33.3%)
Yes	16 (66.7%)
**Diagnosis**	
Astrocytoma, IDH-mutant, WHO Grade 2	2 (8.3%)
Astrocytoma, IDH-mutant, WHO Grade 3	3 (12.5%)
Glioblastoma, IDH-wildtype, WHO Grade 4	16 (66.7%)
Oligodendroglioma, IDH-mutant and 1p/19q-codeleted, WHO Grade 3	3 (12.5%)
**MGMT methylation status**	
Methylated	12 (50.0%)
Unmethylated	11 (45.8%)
Not reported	1 (4.2%)
**Time from uPAR PET/MRI scan to surgery (d)**	
Mean (SD)	4.13 (5.55)
Median [Min, Max]	1.00 [0, 21.0]
**WHO performance status (0–5)**	
0	15 (62.5%)
1	9 (37.5%)
**Surgical treatment during follow up (first line**,** mo)**	
Gross total resection	7 (29.2%)
Subtotal resetion	6 (25.0%)
Biopsy	11 (45.8%)
**Adjuvant treatment during follow up (first line**,** mo)**	
Radiotherapy	9 (37.5%)
Concomitant Radiotherapy and Chemotherapy	13 (54.2%)
None	2 (8.3%)
**PET positive lesion**	
Negative	8 (33.3%)
Positive	16 (66.7%)

### Image Analysis

**Fig. 4:**
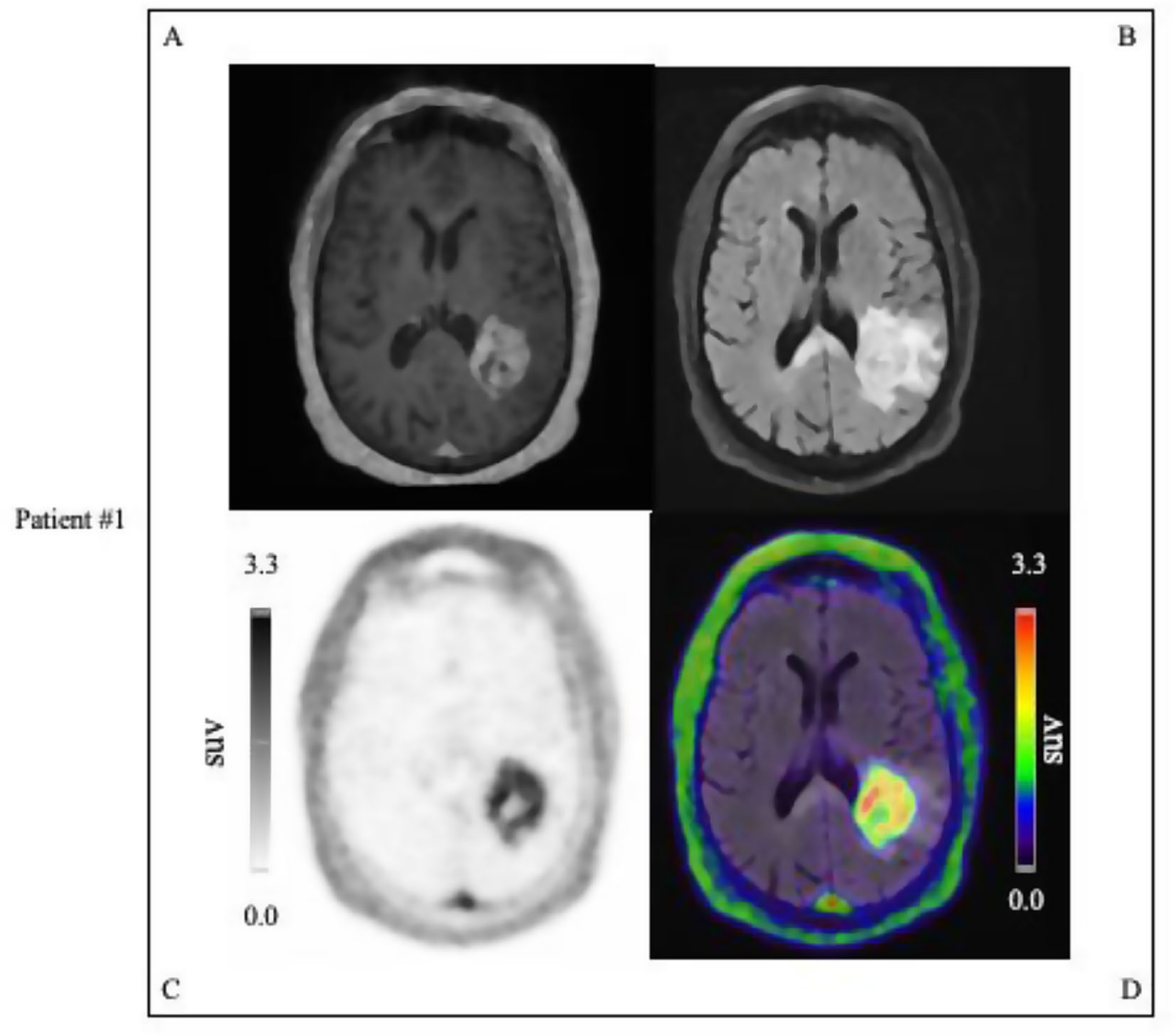
Examples of uPAR PET/MRI performed on patient with glioblastoma, IDH-wildtype, WHO Grade 4 (MGMT non-methylated) in the temporoparietal lobe with tumor SUVmax 3.3: #A T1W MPRAGE MRI with gadolinium contrast, #B T2W FLAIR MRI image, #C uPAR-PET image, #D Merged T2W FLAIR MRI, and uPAR-PET image. Color scale from 0 to tumor SUVmax value of 3.3

**Fig. 5:**
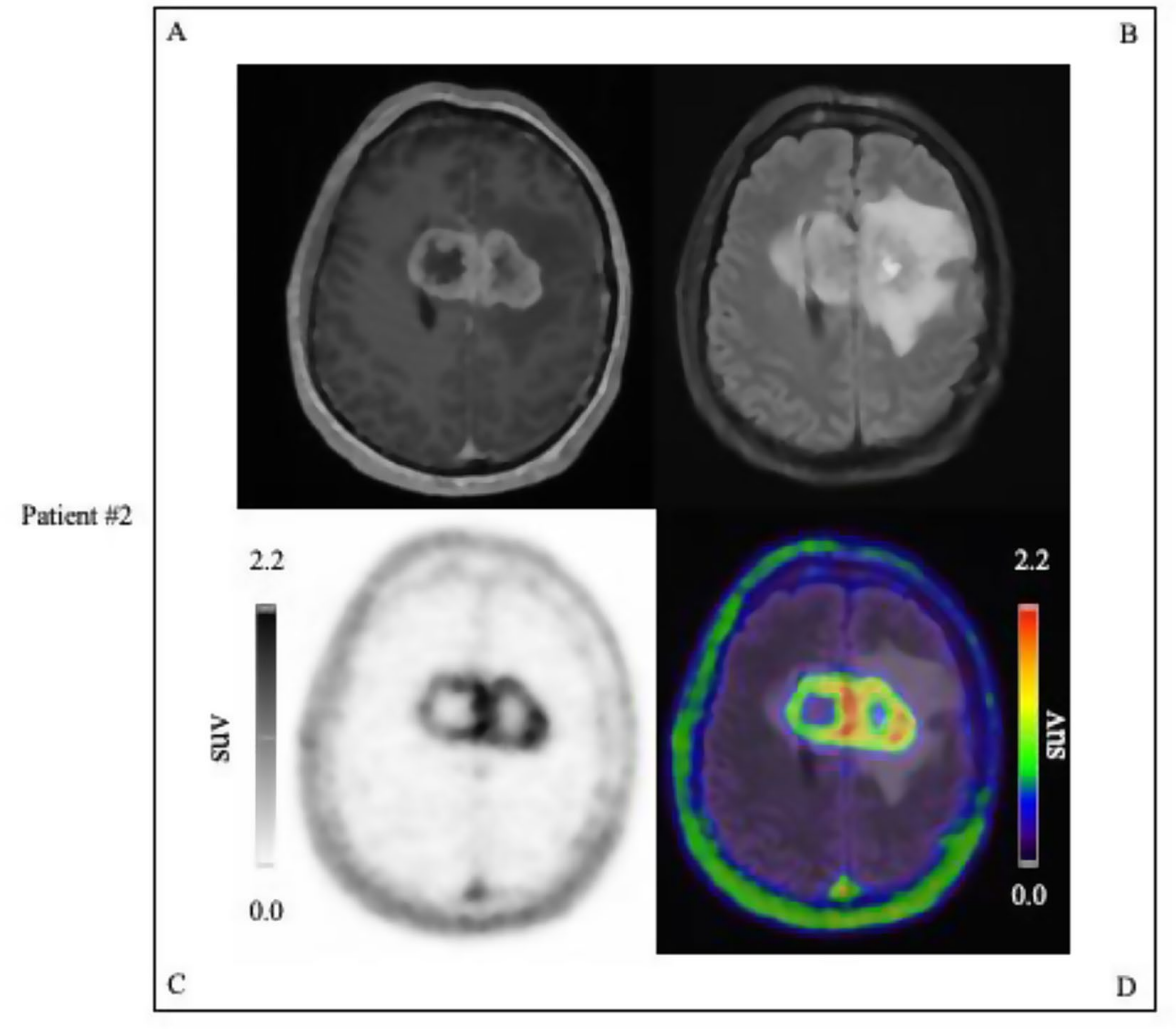
Examples of uPAR PET/MRI performed on patient with glioblastoma, IDH-wildtype, WHO Grade 4 (MGMT non-methylated) involving the genu corpus callosum with tumor SUVmax 2.2: #A T1W MPRAGE MRI with gadolinium contrast, #B T2W FLAIR MRI image, #C UPAR-PET image, #D Merged T2W FLAIR MRI, and uPAR-PET image. Color scale from 0 to tumor SUVmax value of 2.2

Out of the 24 patients, 16 (67%, 16/24) were PET positive. Of the PET positive patients, 15 (94%, 15/16) had contrast enhancement on MRI, whereas one patient (6%, 1/16) had no MRI contrast enhancement (4%, 1/24). Out of the 24 patients, 8 (33%, 8/24) were PET negative. Of the PET negative patients, 8 (100%, 8/8) had no pathological contrast enhancement. Lesions that were uPAR positive were seen primarily among the WHO grade 4 gliomas (94%, 15/16), with one WHO grade 3 glioma patient also presenting a PET positive tumor. Representative examples of PET positive tumor lesions are displayed in Figs. 4 and 5.

### Follow-up

The median follow-up time from uPAR-PET/MRI scan to PFS, OS or when the patients were censored was 18.8 (2.1–45.6) months. A total of 19 (79%) patients experienced disease progression (16 grade 4, 2 grade 3, and 1 grade 2), and 14 (58%) patients died (all grade 4). First-line surgical and oncological treatment in the follow-up period is depicted in Table 2. All patients received surgical treatment, more than half of the patients underwent surgical resection (54%, 13/24), while the rest underwent biopsy (46%, 11/24). The most common oncological treatment was concomitant radio-, and chemotherapy (54%, 13/24), however some patients only received radiotherapy (38%, 9/24) or no adjuvant treatment (8%, 2/24).

### OS and PFS

**Fig. 6:**
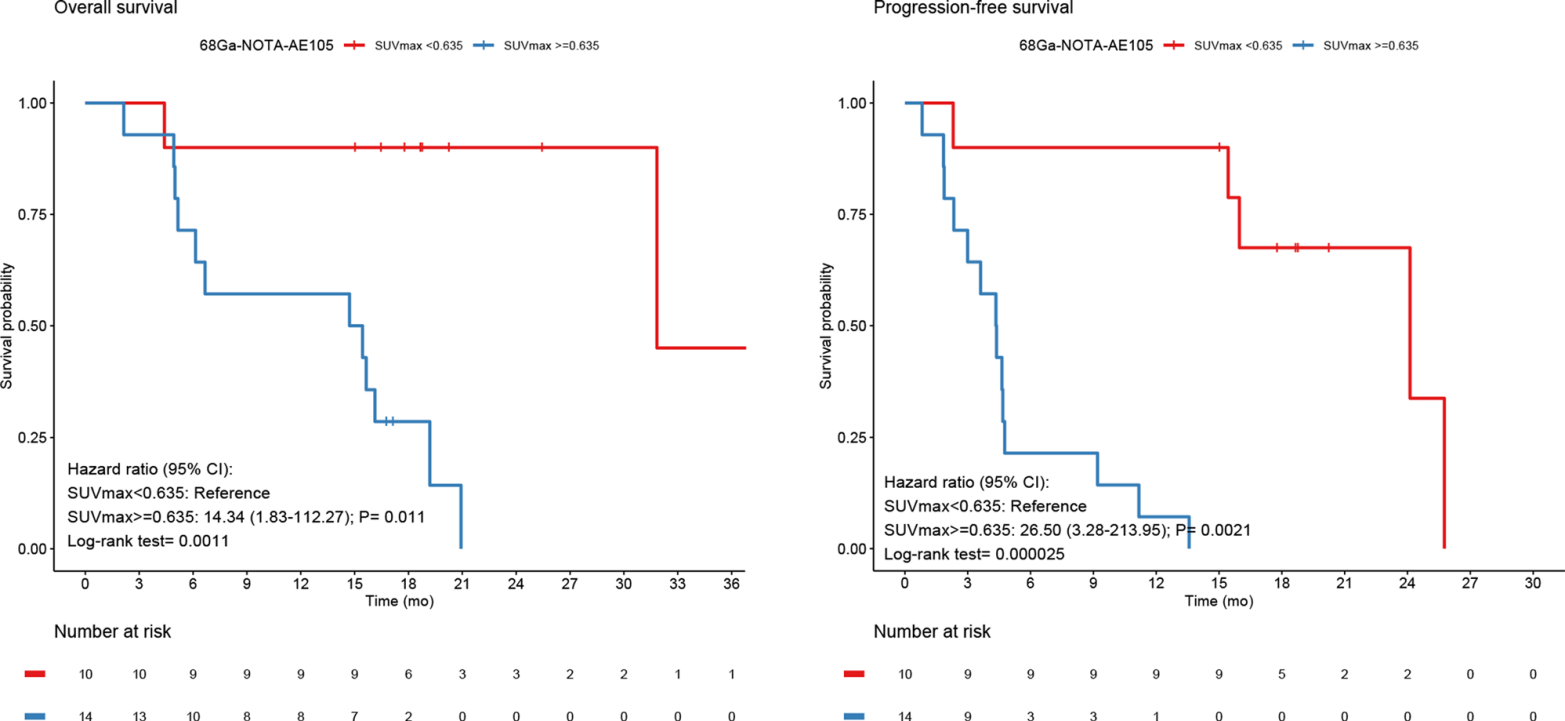
For all primary gliomas Kaplan-Meier Survival plots of OS and PFS dichotomized at SUVmax 0.635

Using the CutoffFinder program, optimal cutoff points for OS and PFS for the group all primary glioma (*n* = 24) by SUVmax were 0.635 both for OS and PFS. Using these cutoffs, uPAR tracer uptake was dichotomized into high and low and revealed a significantly worse prognosis in terms of OS and PFS for patients with high uPAR expression with HR of 14.3 (95% CI, 1.8-112.3; *P* = 0.011; log-rank *P* = 0.0011), and HR of 26.5 (95% CI, 3.3–214.0; *P* = 0.0021; log-rank *P* = 0.000025), respectively (Fig. 6). uPAR expression as a continuous variable was also associated with worse prognosis in terms of OS and PFS with HR of 2.7 (95% CI, 1.5–4.8; *P* = 0.0012), and HR of 2.5 (95% CI, 1.5–4.2; *P* = 0.00073), respectively.

**Fig. 7:**
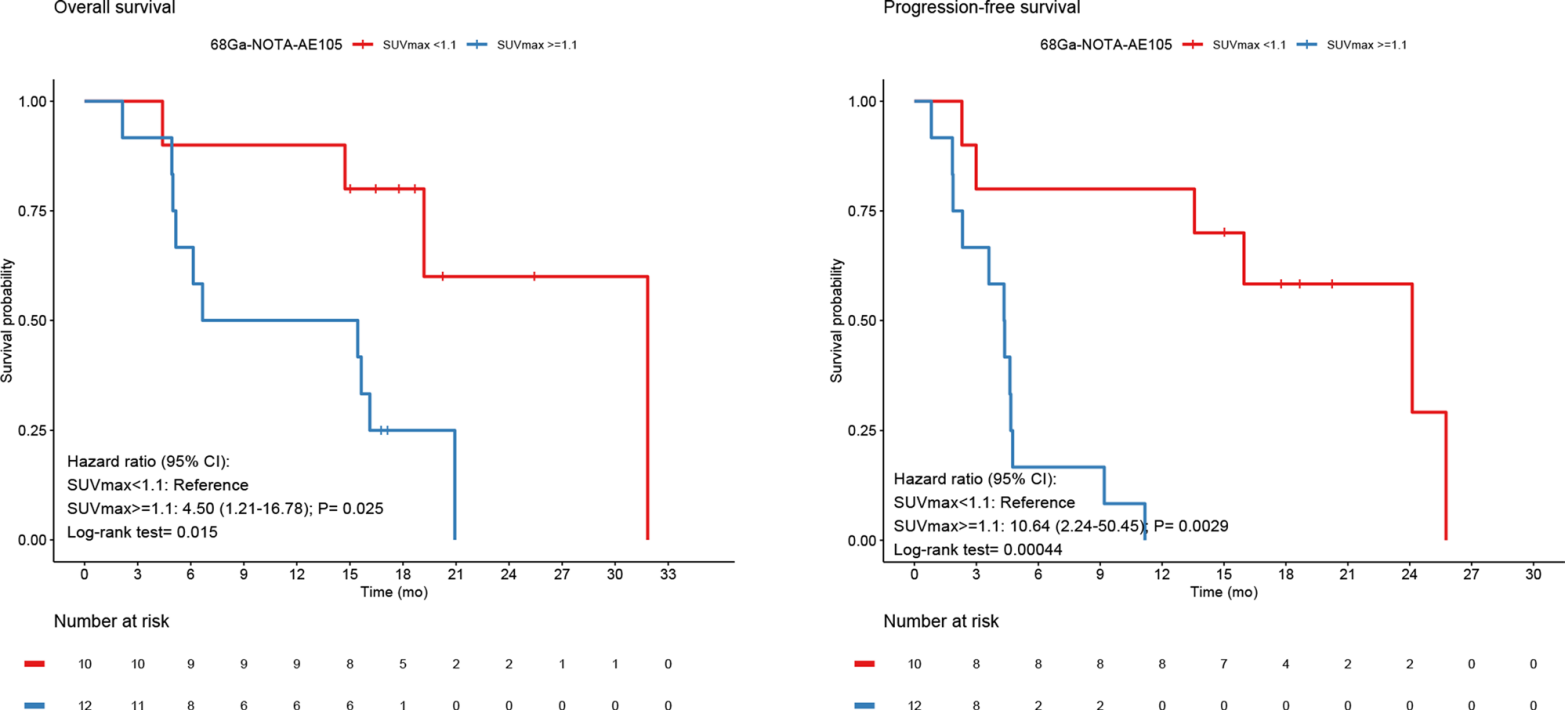
For all primary HGG Kaplan-Meier Survival plots of OS and PFS both dichotomized at SUVmax 1.1

Additional subgroup analysis based on the primary HGGs only (*n* = 22) was also performed. Optimal cutoff point for OS and PFS for the high-grade group by SUVmax was 1.1 for both OS and PFS. uPAR expression dichotomized into high and low also for this subgroup showed significantly worse prognosis for high compared to low uPAR uptake in terms of OS and PFS with HR of 4.5 (95% CI, 1.2–16.8; *P* = 0.025; log rank *P* = 0.015), and HR of 7.4 (95% CI, 2.2;50.5; *P* = 0.0029; log rank *P* = 0.003), respectively (Fig. 7). Furthermore, analysis of uPAR expression as a continuous variable for the subgroup HGG was also associated with worse prognosis in terms of both OS and PFS with HR of 2.4 (95% CI, 1.3–4.4; *P* = 0.0037), and HR of 2.3 (95% CI, 1.4–3.9; *P* = 0.0023), respectively. For Kaplan-Meier analysis and univariate Cox regression analysis for timepoint 40–60 min, please see supplementary material (Supplementary Figs. 1–2, and supplementary Table 1).

## Discussion

In the current study, we found that uPAR-PET activity measured as TumorSUVmax predicted a worse outcome with regard to OS and PFS for patients with primary gliomas. One may attribute this effect to the difference in survival expectancy between the LGG that were uPAR negative and the HGG that constituted the majority of our cohort. However, even when performing the analysis only for HGG uPAR PET was still prognostic. Consequently, uPAR-PET may be used for prognostication and treatment planning, e.g. surgical strategy in these patients. Additionally, we found the majority (67%) of the glioma patients, and in particular almost all HGG patients (94%), to be uPAR-PET positive, which may be encouraging for further development of uPAR-PRRT for use in HGG patients.

Together, these findings highlight the potential of uPAR as a therapeutic target in HGG, and most importantly as a target for uPAR-PRRT. In particular, it should be noted that the positive uptake on uPAR-PET suggests that uPAR-PRRT using a similar ligand, but labeled with a therapeutic alpha or beta emitter, may be administered systemically rather than intratumorally to HGG patients.

It should be noted that external radiotherapy is well established in the treatment of HGG paving the way for targeted radioligand therapy in these patients. PRRT for brain tumors as a highly localized treatment modality is preferable to less precise external radiation therapy as it potentially may reduce the well-known cognitive side effects associated with external radiotherapy due to irradiation of peritumoral margins and normal brain tissue. Established radioligand therapies targeting somatostatin receptors (SSTR-PRRT), primarily used for neuroendocrine neoplasms, have also been pursued in gliomas. The expression of SSTR has been reported in approximately 25% of gliomas with variable expression between LGG and HGG but with decreasing expression of SSTR2 in the most aggressive gliomas [27]. In contrast, we found 94% of WHO grade 4 tumors to be uPAR-PET positive.

PRRT targeting SSTR was investigated in a study where 10 patients with WHO grade 2–3 gliomas were treated with intratumoral injections of ^90^Y-DOTATOC. The ^90^Y-DOTATOC treatment was reported to be both safe and effective in halting tumor progression for at least 13–45 months [28]. Following this, another study demonstrated in a similar fashion the safety and efficacy of ^90^Y-DOTATOC treatment of 3 patients with recurrent glioblastoma in 2010 [29]. In recent years, there has been an increased focus on alpha-emitting PRRT targeting the neurokinin type 1 receptor (NK1R) [30]. Interestingly, PRRT treatment with the alpha-emitting ^213^Bi-DOTA-substance P with intratumoral administration has been demonstrated to be safe in 9 patients with recurrent glioblastomas [31]. Thus, PRRT for gliomas is already under thorough investigation and so far, intratumoral alpha-emitting PRRT has been reported to be safe, feasible, and effective in facilitating clinically meaningful response in several clinical studies underlining the promising role of PRRT as an alternative to conventional therapies against gliomas.

uPAR shows promise for targeted treatment in cancer due to its central role in tumor invasion and metastasis. One reason behind this is the conceptual advantage of targeting a receptor that is predominantly overexpressed in the most aggressive and actively invasive part of the tumors. The data from this study where we found that the majority of the patients displayed uPAR expression and that uPAR expression correlated with worsened outcome, is supported by existing literature where high expression of uPAR, especially in HGG, is found and correlated with poor prognosis [32]. This emphasizes the role of uPAR as a desirable target expressed in the majority of HGG where therapy can be directed towards the most aggressive parts of the tumor, i.e. “dose painting”. Several therapies targeting uPAR have or are currently undergoing investigation but have not been approved for clinical use [33, 34]. Our group published a preclinical paper on uPAR-targeted PRRT with ^177^Lu-DOTA-AE105 treatment of xenografts with colorectal cancer [20]. In this study, we showed a significant reduction of tumor size with good tolerability among the mice. Similarly, we demonstrated the efficacy of ^177^Lu-DOTA-AE105 in treatment in a disseminated metastatic prostate cancer model [21]. Accordingly, PRRT treatment targeting uPAR seems to have a great potential in several tumor types but is yet to be investigated in a clinical setting. An advantage of PRRT treatment with ^177^Lu-DOTA-AE105 is that it is based on the same uPAR binding peptide, AE105, as ^68^Ga-NOTA-AE105 implying the use of uPAR-PET as a companion diagnostic for treatment planning, monitoring, and dosimetry estimation in a uPAR-PRRT theragnostic approach in gliomas.

Although prolonged OS and PFS are the desired objectives of PRRT in patients with gliomas, replacing external radiotherapy may lower the side effects to healthy brain due to more specific tumor tissue targeting.

In this study, we evaluated the prognostic ability of uPAR expression in patients with grade 2–4 gliomas as uPAR expression has been previously been shown to add valuable prognostic information to solid cancers independent of other grading systems such as WHO [17, 35]. However, one limitation of our study is that the majority of patients are diagnosed with HGG and the potential role of uPAR-targeting in LGG is not illuminated to satisfaction. Moreover, the role of the blood-brain barrier (BBB) particularly in LGG, which in most cases are not contrast enhancing is yet to be elucidated. Also, it could be argued that the prognostic value found is mainly driven by whether the BBB is intact or not as reflected by MRI contrast enhancement. Against this conception stands that uptake on uPAR-PET was significant as a continuous variable, i.e. the higher uptake the worse prognosis, both for PFS and OS also when performing the analysis only for HGG patients. Furthermore, it is difficult to evaluate whether the uPAR-PET ligand passes the BBB as the tumors with intact BBB also are less aggressive, and thus would be expected to have a lower uPAR-PET uptake. In preclinical studies where the uPAR-PET binding peptide, AE105, used in our PET tracer was labeled with fluorophores, it has been shown that in an orthotopic GBM model that the tracer seems to reach the diffuse cancer cells outside the bulk tumor (unpublished data) and in a BBB spheroid model, a clear indications of BBB crossing was found [36]. However, we cannot from the present study evaluate whether and to what extent the BBB is a limitation for tracer access and thereby the effect of future uPAR-PRRT. Regardless of this, it should be noted that even if BBB should be a challenge, there are methods to ensure BBB passage by modifying our current ligands. Finally, it could be of interest to study the prognostic value of uPAR-PET in the subgroup of glioblastomas. However, the limited number of patients in this group did not allow us to perform such a subgroup analysis with a relevant statistical power.

## Conclusion

We demonstrate that uPAR expression as measured by uPAR-PET is significantly correlated with a worse outcome for patients with primary gliomas as well as for patients with HGG for both OS and PFS indicating the prognostic value of the radiotracer ^68^Ga-NOTA-AE105. This emphasized uPAR as a promising target for diagnosis, prognostication, and targeted therapy against gliomas and in particular HGG. Most importantly, uPAR holds great potential as a therapeutic target for PRRT treatment where uPAR-PET will serve as a companion diagnostic in a theragnostic approach to preselect patients for uPAR-PRRT. However, future studies are needed to validate this potential.

## Electronic supplementary material

Below is the link to the electronic supplementary material.

Supplementary Material 1

## Data Availability

The datasets generated during and/or analyzed during the current study are available from the corresponding author on reasonable request.

## Abbreviations

^68^Ga-NOTA-AE105: [^68^Ga]Ga-NOTA-Asp-Cha-Phe-D-Ser-D-Arg-Tyr-Leu-Trp-Ser-OH
BBB: Blood-brain barrier
DNOG: Danish Neuro-Oncological Group
DWI: Diffusion-weighted imaging
FET: O-(2-[18 F]fluoroethyl)-l-tyrosine
FLAIR: Fluid-Attenuated Inversion Recovery or Dark-fluid
HGG: High-grade gliomas
LGG: Low-grade gliomas
MRI: Magnetic resonance imaging
NK1R: Neurokinin type 1 receptor
OS: Overall survival
PCV: Procarbazine, lomustine, vincristine regimen
PET: Positron emission tomography
PFS: Progression-free survival
PRRT: Peptide receptor radionuclide therapy
RANO: Response Assessment in Neuro-Oncology
SD: Standard deviation
SSTR-PRRT: Somatostatin receptors
SUVmax: Maximum standardized uptake values
SUVmean: Standardized uptake value
T1W: T1-weighted
T2W: T2-weighted
TBR: TumorSUVmax-to-Normal-BrainSUVmean ratio
TIRM: Turbo inversion recovery magnitude
TMZ: Temozolomide
uPA: Urokinase plasminogen activator
uPAR: Urokinase plasminogen activator receptor
