# Supplementary material for: Prospective phase II trial of [68Ga]Ga-NOTA-AE105 uPAR-PET/MRI in patients with primary gliomas: Prognostic value and Implications for uPAR-targeted Radionuclide Therapy
Source: EJNMMI Res. 2024 Oct 29;14:100. doi: 10.1186/s13550-024-01164-9 (PMC11522270; doi:10.1186/s13550-024-01164-9)
Supplement: Supplementary file 1 — Supplementary Material 1 [file 13550_2024_1164_MOESM1_ESM.docx]

**SUPPLEMENTARY FIGURES AND TABLES**

**FIGURE 1.** For all primary gliomas Kaplan Meier Survival plots of OS and PFS dichotomized at SUVmax 0.69 analyzed at time 40-60 min after tracer injection.

**FIGURE 2.** For all primary HGG Kaplan Meier Survival plots of OS and PFS dichotomized at SUVmax 1.065 analyzed at time 40-60 min after tracer injection.

**
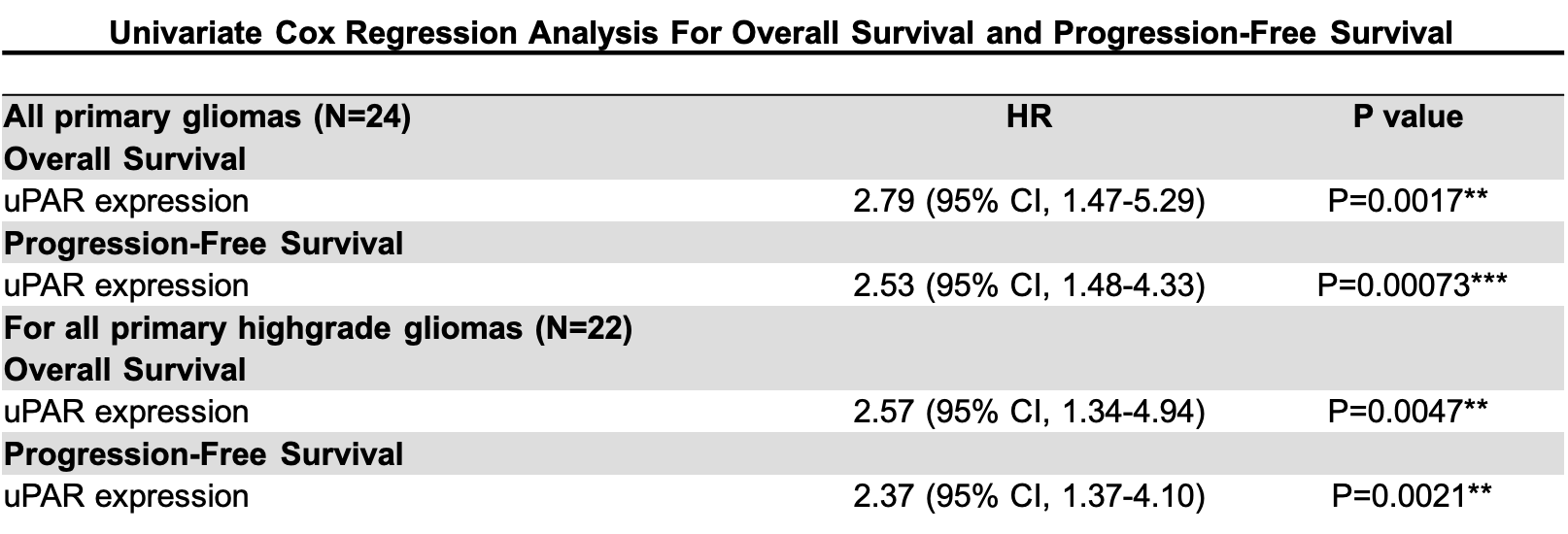
**

**TABLE 1.** Univariate Cox regression analysis performed on the groups all primary glioma (N=24) and HGG (N=22) with uPAR expression as a continuous variable analyzed on images from time 40-60min after tracer injection.
